# Artemisinin Antimalarials: Preserving the “Magic Bullet”

**DOI:** 10.1002/ddr.20344

**Published:** 2010-02

**Authors:** Richard J Maude, Charles J Woodrow, Lisa J White

**Affiliations:** Mahidol-Oxford Tropical Medicine Research Unit, Faculty of Tropical Medicine, Mahidol UniversityBangkok 10400, Thailand; Nuffield Department of Clinical Medicine, Centre for Clinical Vaccinology and Tropical Medicine, University of OxfordOxford, OX3 7LJ, United Kingdom; Department of Infection and Tropical Medicine, Heartlands HospitalBordesley Green, Birmingham, B9 5SS, United Kingdom; Department of Cellular and Molecular Medicine, Centre for Infection, St. George's, University of LondonTooting, London SW17 0RE, United Kingdom

**Keywords:** artemisinin, malaria, resistance, antimalarial

## Abstract

The artemisinins are the most effective antimalarial drugs known. They possess a remarkably wide therapeutic index. These agents have been used in traditional Chinese herbal medicine for more than 2,000 years but were not subjected to scientific scrutiny until the 1970s. The first formal clinical trials of the artemisinins, and the development of methods for their industrial scale production, followed rapidly. A decade later, Chinese scientists shared their findings with the rest of the world; since then, a significant body of international trial evidence has confirmed these drugs to be far superior to any available alternatives. In particular, they have the ability to rapidly kill a broad range of asexual parasite stages at safe concentrations that are consistently achievable via standard dosing regimens. As their half-life is very short, there was also thought to be a low risk of resistance. These discoveries coincided with the appearance and spread of resistance to all the other major classes of antimalarials. As a result, the artemisinins now form an essential element of recommended first-line antimalarial treatment regimens worldwide. To minimize the risk of artemisinin resistance, they are recommended to be used to treat uncomplicated malaria in combination with other antimalarials as artemisinin combination therapies (ACTs). Their rollout has resulted in documented reductions in malaria prevalence in a number of African and Asian countries. Unfortunately, there are already worrisome early signs of artemisinin resistance appearing in western Cambodia. If this resistance were to spread, it would be disastrous for malaria control efforts worldwide. The enormous challenge for the international community is how to avert this catastrophe and preserve the effectiveness of this antimalarial “magic bullet”. Drug Dev Res 71: 12–19, 2010. © 2009 Wiley-Liss, Inc.

**Table:** 

Strategy, Management and Health Policy				
Enabling Technology, Genomics, Proteomics	Preclinical Research	Preclinical Development Toxicology, Formulation Drug Delivery, Pharmacokinetics	Clinical Development Phases I-III Regulatory, Quality, Manufacturing	Postmarketing Phase IV

## DEVELOPMENT INSIDE CHINA

### Discovery and Isolation

Qinghao (‘blue-green herb’) is the Chinese name for a relatively common plant otherwise known as *Artemisia annua* or sweet wormwood. It has been used as a remedy by Chinese herbalists for more than 2,000 years. The earliest known record was in the book *52 Prescriptions*, discovered in the Mawangdui tomb of the Han Dynasty in 168 BC, where it was first described for the treatment of hemorrhoids [Qinghaosu Antimalaria Coordinating Research Group, 1979; Klayman, 1985]. The first treatment with qinghao of disease resembling malaria was described around the second century AD when Zhang Ji, in his text *On Cold Damage*, recommended treating ‘‘fevers with sweating and jaundice’’ with a mixture containing boiled qinghao [Wilcox et al., 2004]. Around 340 AD, in Hong Ge’s *Handbook of Prescriptions for Emergency Treatment*, a cold extraction method of qinghao was described for the treatment of intermittent fevers [Klayman, 1985]. Qinghao has also been mentioned in several later standard Chinese Materia Medica texts as treatment for fever [Qinghaosu Antimalaria Coordinating Research Group, 1979; Hien and White, 1993]; it has also been used for lice, wounds, boils, sores, and convulsions [Hsu, 2006]. Qinghao has remained in common use in Chinese herbal medicine [Weina, 2008] up to the present day.

Formal investigations of the antimalarial properties of qinghao came as a direct result of the Vietnam War. In May 1967, Ho Chi Minh made a request to Zhou Enlai, the then Prime Minister of China, to help provide new treatments for malaria to reduce its already high mortality among North Vietnamese soldiers [Weina, 2008]. In response, the Chinese government initiated a search for new antimalarial compounds in an initiative called ‘‘Office (or Project) 523’’ [Weina, 2008] involving groups of scientists throughout China. This involved a systematic examination of a large number of indigenous plants used in traditional Chinese remedies [Klayman et al., 1985].

The breakthrough came in 1971 when a low-temperature method, similar to Hong Ge's but with diethyl ether in place of water, was used to produce a crude extract from qinghao [Hsu, 2006]. This extract was subsequently shown to possess antimalarial activity in mice infected with *Plasmodium berghei* [Hien and White, 1993]. This was in contrast to the earlier finding of the lack of efficacy of boiled qinghao. These results were first officially reported in March 1972 [Hsu, 2006]. Animal studies showed no evidence of toxicity in rats, cats, or dogs when administered in high daily doses for up to 7 days [Qinghaosu Antimalaria Coordinating Research Group, 1979]. The active ingredient of qinghao was isolated in 1972 [Hien and White, 1993], as a colorless, needle-shaped crystal. In 1975, the name *qinghaosu* (‘active principle of qinghao’) was chosen for this new compound along with the more Western-sounding “artemisinine,” later changed to “artemisinin” [Klayman et al., 1985]. The molecular formula for artemisinin (C15H22O5), was derived from the results of high-resolution mass spectrometry and elemental analysis, following which the structure was determined by spectral analysis, chemical reactions, and X-ray diffraction. It was shown to be a new type of sesquiterpene lactone with a peroxy group [Qinghaosu Antimalaria Coordinating Research Group, 1979]. By 1973, a method had been found to convert artemisinin to its more active metabolite, dihydroartemisinin (DHA) [Hsu, 2006], and subsequently from DHA to a large number of other derivatives [Haynes and Vonwiller, 1994]. The endo-peroxide moiety was found to be essential for antimalarial activity, and substitutions at the lactone carbonyl group were found to increase potency [Hien and White, 1993]. Industrial replication of these processes, including reduction with sodium borohy-dride to convert artemisinin to DHA, provided the first opportunity for the large-scale production of artemisinin derivatives from plant materials. Two derivatives in particular attracted interest, as they were more soluble and much more active than artemisinin: (1) the oil-soluble ether derivative, artemether, produced in Kunming; and (2) the water-soluble hemiester derivative, artesunate, produced in Guilin [Haynes and Vonwiller, 1994]. Complex methods have been described for the total synthesis of artemisinin and its derivatives [Zhou, 1986], but these are not cost effective for large-scale production [Klayman et al., 1985]; thus, the artemisinins continue to be of plant origin.

### Development as Monotherapy

In 1972, 21 patients with *Plasmodium falciparum* and *vivax* malaria were successfully treated using the diethyl ether extract of qinghao in Beijing [Hsu, 2006]. Different extractions of qinghao were used successfully from 1974-1978 in a large multicenter trial of 2,099 patients with malaria (1511 of these had *P. vivax* and 558 *P. falciparum*, including 143 with chloroquine resistant, and 141 with cerebral, malaria) [Qinghaosu Antimalaria Coordinating Research Group, 1979]. Artemisinin produced a rapid clinical cure (clearance of fever and asexual parasites in the blood) in almost all patients, without obvious toxicity, and a faster parasite clearance than quinine in cerebral malaria. However, there were high recrudescence rates of around 10-25%.

It was not until 1979 that Chinese scientists first published clinical findings for the artemisinin anti-malarials in an international scientific journal, the *Chinese Medical Journal*, in a paper entitled ‘‘antimalarial studies on qinghaosu’’ [Qinghaosu Antimalaria Coordinating Research Group, 1979]. This paper summarized much of what was known about the chemistry, pharmacology, and clinical efficacy of the artemisinins including the results of in vitro studies, animal studies, and human trials, and was the first time this new group of antimalarials came to the attention of the West. At that time, however, China was a relatively closed country, and there was great skepticism in the West that a previously unknown natural remedy could be so effective [Weina, 2008]. Furthermore, manufacturers of artemisinin drugs in China did not meet international standards for Good Manufacturing Practice (GMP) [Wright, 2002]. Bodies such as the World Health Organization (WHO) were not sufficiently convinced by the available research results to recommend the artemisinins at that time for the treatment of malaria.

Since 1979, a number of other Chinese clinical trials investigated the efficacy of oral, intramuscular, and intravenous artesunate, intramuscular artemether, and oral dihydroartemisinin. Very little of this trial data has been published outside of China, one exception being studies by a group in Guangzhou. In total, artemisinins were administered to 2,150 cases with *P. falciparum* (including 91 with severe disease), and 105 cases of *P. vivax* malaria. These compounds were found to be rapidly effective with recrudescence rates of 5-10% following 5- to 7-day treatment courses and there was little evidence of toxicity [Li et al., 1994].

In October 1981, at the Fourth Meeting of the Scientific Working Group on the Chemotherapy of Malaria of the WHO, in Beijing, Chinese researchers summarized the research results to date on the antimalarial qinghaosu and its derivatives. WHO representatives were very impressed by what they heard. They approached the Chinese government for samples of the plant and details of the extraction techniques to verify the Chinese findings. However, the Chinese representatives were reluctant to share these details and further collaboration did not occur at that time [Qigui et al., 2007].

## DEVELOPMENT OUTSIDE CHINA

### Confirmation and Toxicity Studies

As a result of the caution expressed by the Chinese, a group of scientists from the U.S. Army's Division of Experimental Therapeutics, Walter Reed Army Institute of Research (WRAIR), began a search for the plant in the United States. They attempted to replicate the extraction of artemisinin from *Artemisia annua* serendipitously discovered growing in Virginia and near Washington DC, supplemented by material from the U.S. National Arboretum. In 1984 they finally succeeded and published their findings, including its extraction with petroleum ether, chromatographic separation, and potent antimalarial activity against chloroquine sensitive and resistant strains of *P. falciparum* in vitro [Klayman et al., 1984]. During the following years, much of the earlier Chinese work was replicated and the remarkably high efficacy of the artemisinins against malaria in vivo was confirmed. In Thailand and Vietnam, a number of studies conducted between 1987 and 1994 also confirmed the Chinese findings of high efficacy and low toxicity [Hien, 1994; Looareesuwan, 1994]. However, a series of animal experiments by another group at the WRAIR, using artemotil (arteether) and artemether and published in 1994, showed that these drugs could produce fatal neurotoxicity in high dose, causing brain damage in rats and dogs [Brewer et al., 1994]. This finding caused some organizations to lose interest in these compounds. However, this neurotoxicity has since been shown not to occur with the water-soluble derivatives, such as artesunate, and no correlate of the animal pathology has been reliably demonstrated in humans [White, 2008].

### Treatment of Severe Disease

Parenteral artemether has to be given by intramuscular injection, as it is soluble in oil but not in water. Its bioavailability is thus more variable than intravenous artesunate due to its slow and erratic absorption, and it is perhaps not surprising that it is a less effective antimalarial, particularly in severely ill patients. However, several randomized trials during the 1990s and early 2000s showed it to be equally efficacious to intravenous quinine [Artemether-Quinine Meta-analysis Study Group, 2001], although a lack of superiority over quinine hindered its further development.

Because of its solubility in water, artesunate is the only compound that is suitable for intravenous injection for use in patients with severe disease. In 2005, the results of a large multinational antimalarial treatment trial of severe malaria were published, the SEAQUA-MAT study [Dondorp et al., 2005]. This was a randomized trial in predominantly Asian adults of intravenous quinine versus intravenous artesunate. This trial found a 34.7% lower mortality in patients who received artesunate rather than quinine. As a result, the 2006 WHO Guidelines [World Health Organization, 2006] were changed to include, for the first time, intravenous artesunate as the recommended first-line treatment for severe malaria. This replaced quinine for low transmission settings and was recommended as an alternative to quinine for children in high transmission settings. Initially, due to concerns about possible toxicity in humans, based on the previous animal studies, the dose of intravenous artesunate was kept low, but in this guideline it was doubled to 2.4mg/kg. There was no evidence of neurotoxicity in the SEAQUAMAT study, and a subsequent detailed study showed no effect of intravenous artesunate on the heart [Maude, 2009a]. A subsequent multicenter trial based on the SEAQUAMAT design is currently underway in African children [Day and Dondorp, 2007].

Rectal preparations of artemisinins are also available for situations where parenteral therapy is not immediately available; a single dose of pre-referral rectal artesunate has recently been shown to reduce mortality when access to injections will take several hours [Gomes et al., 2009].

### Combination Therapy

Two disadvantages of the artemisinins when used as monotherapy are the relatively high recrudescence rate of around 10% and the need for a 7-day course (with associated poor compliance) in order to achieve radical cure [White, 2008]. One solution to these problems is to use the drug in combination with a second antimalarial, the so-called artemisinin combination therapies (ACTs). With ACTs, the duration of treatment is only 3 days and the exposure of parasites to artemisinin monotherapy is minimized, reducing the likelihood of artemisinin resistance.

Because of spreading mefloquine resistance around the Thai-Burmese border area, artesunate-mefloquine ACT was introduced as a replacement for mefloquine monotherapy in Thailand during the early 1990s [Nosten et al., 1994]. This was the first large-scale use of an ACT in the field. A series of clinical studies showed this new regimen to have extremely high cure rates, to have successfully halted the spread of mefloquine resistance and reduced the incidence of malaria [Nosten et al., 2000]. It continues to be used there today as two separately formulated medications, and a fixed dose combination has recently become available.

Chinese researchers at the Beijing Academy of Military Medical Sciences during the 1980s combined oral artemether with another drug they had developed, benflumetol (lumefantrine), as a treatment for non-severe malaria. In 1990, Chinese officials came to an agreement with Swiss pharamaceutical company No-vartis (then Ciba-Geigy) to work together to develop, test, and manufacture this artemisinin combination therapy, later called Coartem. Novartis assisted their Chinese partners to redesign local production facilities, upgrade their quality assurance systems, and construct new factories to ensure compliance with GMP standards. Novartis acquired the rights to market the therapy outside China in 1994, although Chinese companies continue to supply the raw material for the drug to Novartis, and China continues to hold a domestic patent for the therapy. Today, Coartem is produced by Novartis in China and the United States and is the most widely used artemisinin combination worldwide. Food and Drug Administration (FDA) approval was given for use of artemether-lumefantrine in the United States in April 2009. This ACT has the advantage of being one of few coformulated ACTs to be produced to international GMP standards, and it is therefore on the WHO list of pre-qualified medications. Coformulation means both drugs are combined in the same tablet; this is designed to prevent patients from taking only the artemisinin component to avoid side effects from the partner drug, a particular problem with artesunate-mefloquine. The other major coformulated ACT is dihydroartemisin-piperaquine (DHA-PQP), and this has been shown to be a safe, well tolerated, and highly effective treatment of *P. falciparum* malaria in Asia and Africa [Zwang et al., 2009]. DHA-PQP has not yet been confirmed to be manufactured in accordance with GMP standards, although prequalifica-tion is currently under way. Hence, artemether-lumefantrine remains the drug of choice recommended by the WHO for uncomplicated malaria.

Together with their partners, the Drugs for Neglected Diseases initiative have recently developed two other coformulated ACTs: artesunate-amodiaquine (ASAQ/Coarsucam) and artesunate-mefloquine (ASMQ). ASAQ was introduced in 2007 and joined artemether-lumefantrine as the only other WHO prequalified ACT. It has the advantage of once a day administration and is now available in 24 countries in sub-Saharan Africa. ASMQ was first used in Brazil in 2008 and will be introduced to other countries in South America and Southeast Asia over the next few years.

Oral ACTs have been shown in a number of clinical trials over the past decade to be the most effective antimalarials for uncomplicated disease [Mayxay et al., 2004; Stohrer et al., 2004; Guthmann et al., 2005; van den Broek et al., 2005; Piola et al., 2005; Falade et al., 2005; Stepniewska et al., 2004; Mutabingwa et al., 2005]. A large body of clinical and trial experience has also reassured the international community of the very low toxicity of the artemisinins. Because of concerns about resistance having arisen to all major classes of antimalarials other than the artemisinins, and the desire to preserve the effectiveness of the artemisinins, the WHO chose to switch policy in 2004 to recommend ACTs as treatment for malaria in areas where resistance to antimalarial monotherapies had arisen (chloroquine, sulfadoxine/ pyrimethamine, and amodiaquine) [Roll Back Malaria, 2004]. In 2006, they became recommended first-line treatment for uncomplicated *P. falciparum* malaria worldwide [World Health Organization, 2006].

### Availability of Artemisinins

By May 2008 only 11 countries had not yet adopted ACT for first-line treatment of uncomplicated disease due to *P. falciparum* [World Health Organization, 2008a]. Unfortunately, there are still few production facilities approved to international GMP standards that can manufacture ACTs so that demand continues to exceed supply. There have also been problems with falsely inflated purchase costs, fake artemisinins in Asia [Dondorp et al., 2004; Newton et al., 2006], and Africa and poor quality control within certain batches of drug. In addition, available supplies of these drugs have been insufficient to meet demand in the developing world; thus, market prices of ACTs are many times higher than the now almost useless chloroquine and sulfadox-ine-pyrimethamine [Woodrow et al., 2005]. New initiatives by the Global Fund, WHO, Gates Foundation, and Roll Back Malaria, among others, to encourage the increase in production and subsidize costs are helping to remedy this situation. There are also a number of new synthetic artemisinin derivatives and ACTs currently under development [Olliaro and Wells, 2009].

The vast majority of intravenous artesunate is still produced in China and Vietnam and it is not widely available elsewhere [Anstey et al., 2006]. The major hurdle is that none of the production facilities in these countries are GMP-certified and thus cannot be licensed by many national bodies [Anstey et al., 2006]. It is also difficult to secure sufficient quantity of supply. Only Brazil, China, Iran, Lao, Myanmar, Papua New Guinea, Solomon Islands, Thailand, and Vietnam have so far adopted artesunate as national treatment policy for severe malaria [World Health Organization, 2008c]. In 2006, intravenous artesunate replaced quinine as first-line treatment for severe disease in the national Therapeutic Guidelines of Australia, where it remains unlicensed but is available under a Special Access Scheme [Anstey et al., 2006]. It is also available on a named patient basis in the United Kingdom and United States. Until GMP-certified artesunate becomes more available, this situation is unlikely to change.

## ARTEMISININ RESISTANCE

Antimalarial resistance can be detected as reduced in vivo or in vitro responses, or via use of molecular markers proved to mediate drug resistance. Because artemisinins are used predominantly in combination with other drugs (as ACTs), measures of parasite clearance are more sensitive markers of reduced susceptibility than recrudescence rates (which are influenced strongly by resistance to the partner drug). Until recently, there has been no evidence for a significant reduction in artemisinin efficacy at either clinical or in vitro levels. In broad terms, the rapid antimalarial action of artemisinins, combined with their pharmacokinetic properties (with terminal elimination half-lives in the order a few hours), led to the belief that resistance to artemisinins would be very slow to develop since exposure of parasites to subtherapeutic levels of drug would be very brief. However, in 2006, the WHO issued an ultimatum to providers to stop selling artemisinins as monotherapy to reduce the risk of resistance arising and increase the useful lifespan of this class of antimalarials [Nelson, 2006]. In fact, artemisinins had already been widely available as monotherapy for many years in a few countries. In Cambodia, artemisinin monotherapies were first introduced from China more than 30 years ago [White, 2008]. For most of the period since then, they have been available through the private sector in subtherapeutic doses due to inappropriately abbreviated treatment courses [Yeung et al., 2008], poor quality manufacture, or combination with fake drugs [Newton et al., 2006]. Thus many malaria sufferers in Cambodia received artemisinin monotherapies that failed to cure their infection. This combination of continuously multiplying parasites in the presence of nonlethal drug levels produces ideal conditions for the generation of artemisinin resistance [Stepniewska and White, 2008; Maude et al., 2009b; Pongtavornpinyo et al., 2009]. Cambodia was one of the first countries to switch first-line treatment to ACT in national antimalarial drug policy (artesunate-mefloquine in 2000) [Yeung et al., 2008], and a blanket ban on the use of artemisinin monotherapies has recently been introduced there.

Since 2003, there have been isolated reports of high failure rates and reduced in vitro responses to artemisinins in parts of Asia [Vijaykadga et al., 2006; Yang et al., 2003; Jambou et al., 2005; Denis et al., 2006a,b]. In December 2008, it was reported that there were prolonged parasite clearance times, despite adequate drug levels, in 2 out of 60 cases of falciparum malaria in a study in Cambodia [Noedl et al., 2008]. This was followed in mid-2009 by a detailed report describing markedly prolonged time to parasite clearance in patients treated with artesunate in Pailin, Cambodia [Dondorp et al., 2009]. The reduced parasitologic response could not be explained by pharmacokinetic or other host factors. The most likely explanation for this very concerning finding is infection with *P. falciparum* that is to some degree resistant to artesunate. Inappropriate use of artemisinin monotherapies is probably at least partly responsible for this resistance having arisen first in Cambodia, particularly as they have been available for so long there. This practice is not unique to Cambodia [Burki, 2009], however, and the lack of any robust signs of resistance elsewhere suggests that there are likely to be other contributory factors. If no action is taken, it is likely that this mildly resistant phenotype will become increasingly resistant and ultimately spread elsewhere. Previously, chloroquine and sulfadoxine pyrimetha-mine-resistant malaria first arose in Cambodia and subsequently spread across the world [Roper et al., 2004; Verdrager, 1986]. With the ongoing increase in ease of traveling between distant places, the potential for rapid spread of such phenotypes is greater now than it has ever been. By negating the hugely beneficial contribution of artemisinin combination therapies, artemisinin-resistant malaria, wherever it appears, would be a disaster for malaria control and elimination efforts.

Molecular detection of artemisinin resistance has remained compromised by the fact that the mechanism of action of this group of compounds is still not well understood. The organellar location of their antimalar-ial action, and the physical nature of their target and how they interact with it, represent some of the greatest controversies in current malaria biology [Fidock et al., 2008]. Amplification of the *P. falciparum* multidrug resistance gene *Pfmdr1* is associated with increased IC50 values for artemisinins [Price et al., 2004], but the effect is small and is clearly not associated with reduced in vivo susceptibility; parasite clearance rates remain unchanged in parasites with multiple copies of *Pfmdr1.*

## PRESERVING THE MAGIC BULLET

To avoid the doomsday scenario of global spread of artemisinin resistance, there must be urgent intervention in Western Cambodia. In response to the preliminary results from Pailin and elsewhere, the WHO convened a series of meetings to discuss how to proceed. It was agreed that containment of artemisinin resistance would involve removing selection pressure and reducing and ultimately eliminating falciparum malaria in Western Cambodia. Strategies being considered include prohibiting the use of artemisinin monotherapy and introducing an alternative, such as dihydroartemisinin-piperaquine or atovaquone-progua-nil, as treatment or in a mass screen-and-treat campaign, in combination with insecticide treated bednets [World Health Organization, 2008b,c,d]. In the absence of sufficient data and time in which to collect it, mathematical modeling is being employed to help inform these decisions [Maude et al., 2009b,c].

## CONCLUSION

Currently there is a paucity of promising novel antimalarial drugs under development and a loss of the artemisinins to resistance would be a disaster for international malaria control. With the most effective and least toxic antimalarial known still in our arsenal, in combination with far more effective vector control measures than ever before, we currently have the best opportunity in history to eliminate-and, it is hoped, eradicate-malaria. It would be a tragedy if we were to squander this opportunity by failing to preserve the artemisinins as a ‘‘magic bullet’’ against malaria.
